# Determining Protein Complex Connectivity Using a Probabilistic Deletion Network Derived from Quantitative Proteomics

**DOI:** 10.1371/journal.pone.0007310

**Published:** 2009-10-06

**Authors:** Mihaela E. Sardiu, Joshua M. Gilmore, Michael J. Carrozza, Bing Li, Jerry L. Workman, Laurence Florens, Michael P. Washburn

**Affiliations:** 1 Stowers Institute for Medical Research, Kansas City, Missouri, United States of America; 2 Laboratory of Structural Biology, National Institute of Environmental Health Sciences, Research Triangle Park, North Carolina, United States of America; 3 Department of Molecular Biology, UT Southwestern Medical Center, Dallas, Texas, United States of America; University of Southampton, United Kingdom

## Abstract

Protein complexes are key molecular machines executing a variety of essential cellular processes. Despite the availability of genome-wide protein-protein interaction studies, determining the connectivity between proteins within a complex remains a major challenge. Here we demonstrate a method that is able to predict the relationship of proteins within a stable protein complex. We employed a combination of computational approaches and a systematic collection of quantitative proteomics data from wild-type and deletion strain purifications to build a quantitative deletion-interaction network map and subsequently convert the resulting data into an interdependency-interaction model of a complex. We applied this approach to a data set generated from components of the *Saccharomyces cerevisiae* Rpd3 histone deacetylase complexes, which consists of two distinct small and large complexes that are held together by a module consisting of Rpd3, Sin3 and Ume1. The resulting representation reveals new protein-protein interactions and new submodule relationships, providing novel information for mapping the functional organization of a complex.

## Introduction

Most proteins exert their function together with other proteins by forming distinct complexes which are responsible for specific processes in a cell. Therefore, knowing how proteins associate into stable protein complexes is an essential part of understanding cellular activity. Proteins within each complex can be distinguished as different classes, and therefore are designated as core (always present in each isoform of a complex), module (shared functional subunits of different complexes) or attachment proteins (present only in some purifications) [1], [2]. We recently demonstrated that quantitative proteomics can be used to separate the proteins in those three classes as well as to generate probabilistic protein interaction networks that provide the degree of association between proteins within the respective protein complexes [3]. However, most protein complexes are stable complexes in which the proteins are recovered at equal abundance levels, hence determining the connectivity between proteins in those complexes solely from quantitative proteomics experiments remains a major challenge.

To address this task, we developed a new approach and applied it to a dataset aimed to characterize the histone deacetylase (HDAC) Rpd3 complexes in *Saccharomyces cerevisiae*. Histone deacetylation is a critical process in transcriptional regulation and Rpd3 is known to be involved in both activation and repression of transcription [4]. The yeast HDAC Rpd3 is a homologue of Class I human HDACs and is known to function in two separate complexes termed Rpd3 Small(S) and Rpd3 Large(L) [5], [6]. The proteins in these complexes were recently defined. The Rpd3S complex, which suppresses spurious intergenic transcription initiation [5], consists of Eaf3, Rco1, Rpd3, Sin3 and Ume1 [6], [7]. Rpd3, Sin3 and Ume1 have also been shown to belong to the Rpd3L complex along with additional components that are Pho23, Sds3, Sap30, Dep1, Rxt2, Rxt3, and Cti6 [5]. The sequence specific repression proteins, Ash1 and Ume6 are also stably integrated into the Rpd3L complex [5]. In addition, HDAC inhibitors have emerged as important therapeutic targets for the treatment of cancer and other human diseases [8].

Despite the importance of the Rpd3 complexes, little is known about the interactions of proteins within the complexes. Here we show that quantitative proteomics coupled with hierarchical clustering analysis and probabilistic methods can be used to build a deletion-interaction network and suggest a model of a multiprotein complex. Although a quantitative analysis of the intact complex was not sufficient, we show that a systematic comparison of quantitative deletion strain purifications to its wild-type counterpart allowed us to predict the interactions between the proteins within the Rpd3 complexes. We developed computational methods to calculate these interactions from which we assembled an interdependency-interaction model of these important regulatory complexes.

## Results

### Data generation for the wild-type histone deacetylase complex

A total of 11 different Rpd3 subunits were TAP-tagged (hereafter referred to as “baits”), expressed and purified by affinity purification, and analyzed by multidimensional protein identification technology (MudPIT) [9], [10] leading to the identification of 534 non-redundant (NR) proteins (Table S1). Relative protein levels were estimated by calculating distributed normalized spectral abundance factors (dNSAF) [11]. The non-specific binders were extracted from the dataset by comparing the dNSAF value in each of the individual purifications with the dNSAF value from the negative control (Figure S1). If the dNSAF value in the purification was lower than the dNSAF in the negative control, the protein was considered non-specific to that particular purification and the dNSAF was replaced by 0, otherwise the dNSAF value remained unchanged. After removing the proteins shown to be non-specific to all 11 purifications, 429 proteins remained for further analysis. Next, to reduce the dataset to the most information rich group of proteins we applied singular value decomposition (SVD) to the dataset, as described previously [3] (Figure S2). The resulting 80 proteins remaining after cut-off included all previously reported members of the Rpd3 complexes, components of the NuA4 complex, eight components of the CCT ring complex, and other proteins. The meaning of the remaining proteins are discussed in the supporting information and provided in Table S5, and the baits were clustered using the Jaccard index (Figure S3) and supporting information.

### Quantitative analysis of wild-type and deletion purifications

**Figure 1:**
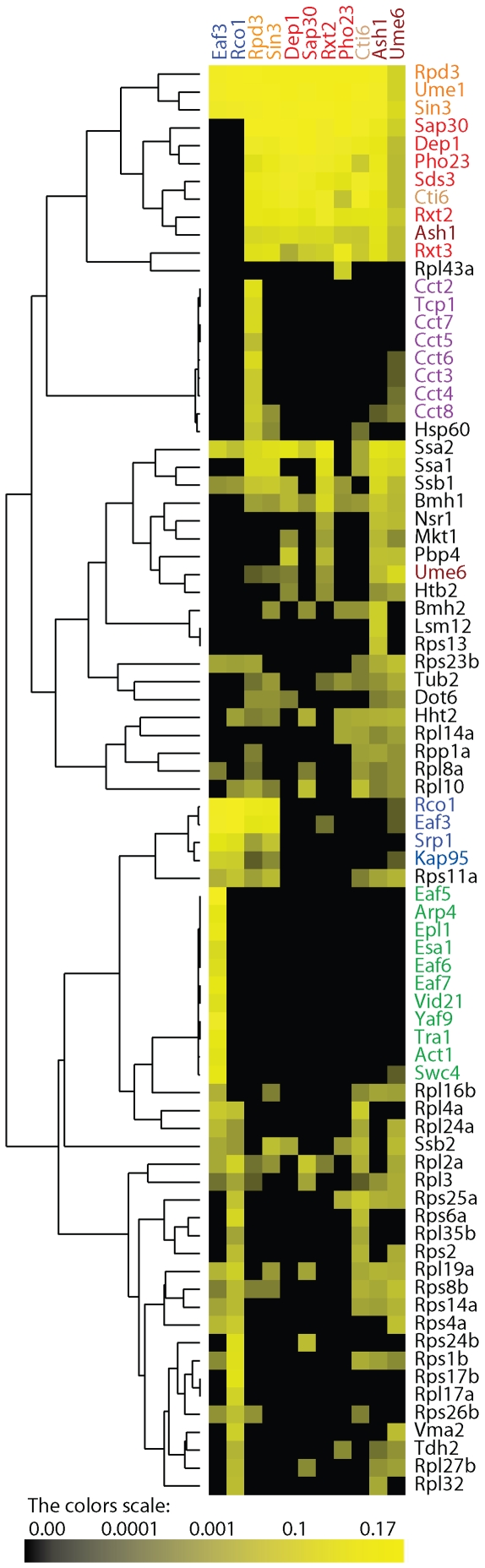
Hierarchical clustering on the wild-type purifications. A hierarchical cluster was performed on the relative protein abundances expressed as dNSAFs. Each *column* represents an isolated purification, and each *row* represents an individual protein (prey). The color intensity represents protein abundance with the brightest yellow indicating highest abundance and decreasing intensity indicating decreasing abundance. Black indicates that the protein was not detected in a particular purification. The components of the RPD3L are colored in red, the components of the RPD3S complex are colored in blue, Ash1 and Ume6 are colored in dark red, and Cti6 is colored in light brown. The shared module is colored in orange.

To group proteins based on relative abundance level, we performed hierarchical cluster analysis on the 11 baits and 80 preys (Figure 1). The resulting cluster showed that the core components of the large and small complexes were well separated (Figure 1, depicted in red and blue). Proteins that belong to the shared module (orange) were placed into the same branch of the tree as shown by the dendogram and they were positioned adjacent to the large complex. Interestingly, additional proteins (Srp1, Kap95, Hht2, Dot6, and Bmh1) that were not previously characterized as subunits of the RPD3 complexes were detected and placed in close proximity with the known core components of the complexes suggesting their strong association.

**Figure 2:**
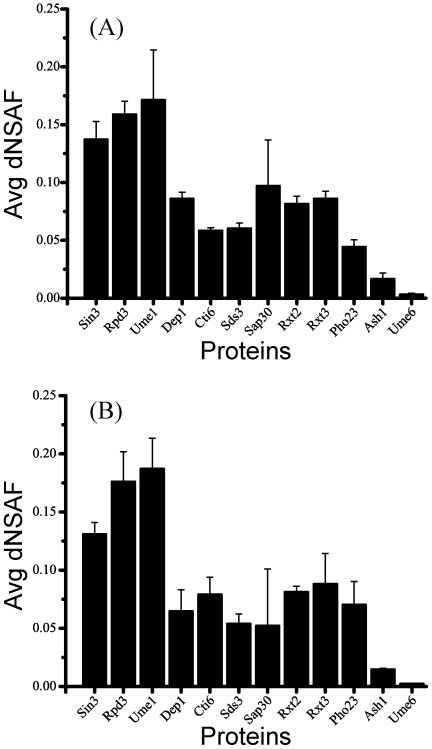
Relative abundances of Rpd3/Sin3 components. For visualization purposes, the components of the Rpd3/Sin3 complexes were represented using (A) Pho23-TAP, and (B) Sap30-TAP, MudPIT analysis was performed on three replicates for each of the purification and the observed dNSAF values for each subunit averaged. Error bars are shown and represent one standard deviation of the data.

We next tried to address whether our analysis is affected by the stoichiometry of a complex. Based on the average dNSAF values in each of the TAP purifications we estimated the stoichiometry of the RPD3 complexes [12] (Figure 2). For the Rpd3L complex, we observed a 2∶1 ratio of dNSAF values for the module proteins relative to the other components whereas in the case of Rpd3S, the dNSAF values of both module proteins and the other subunits were at similar levels suggesting a 1∶1 ratio in the small complex.

These results also show that one component of the large complex, Ume6, was recovered at a substoichiometric levels with the core components of the large complex (Figure 2), hence it was positioned apart from either the large or small complex in the clustering result. We next applied the Bayes' theorem to calculate the posterior probabilities which determine the preference between a prey and a particular bait relative to all other baits [3]. Thereby preys that appear at similar relative abundance levels in all baits receive similar moderate probabilities, independent of their higher or smaller relative abundance level. For example, module proteins, which have the highest relative abundance in the dataset, still have similar posterior probabilities as stable core proteins (Table S4). On the other hand, preys that vary in abundance between the baits will yield higher or lower probabilities respectively, depending on its relative change to all baits. Consequently, the probabilities emphasize the relationship of two proteins in a complex independent of its stoichiometry. While this analysis is able to determine *bona fide* protein interactions and to separate the subunits of the two complexes, on stable complexes like Rpd3 all probabilities in the wild-type network have similar values, therefore reflecting the stability of the complex as a whole and not the pairwise relationship between the proteins.

Instead, we took advantage of a proteomics dataset generated from systematic deletion mutants, a similar approach to those used previously [13], [14]. For example, Mitchell *et al.* confirmed that when used in combination with deletion strains, TAP purification experiments are sufficient to negate interactions [13]. In addition, Collins *et al.* developed a method for calculating an interaction score for protein pairs based upon the presence and absence of the corresponding proteins in affinity purification mass spectrometry experiments [15]. These studies demonstrate that the absence of preys in affinity purification mass spectrometry experiments can be used to generate novel insights into protein complexes.

In the current study, individual components of the Rpd3 complexes are deleted from the genetic background of a strain in which Rpd3 is TAP tagged, resulting in a series of deletion mutants. The first data set includes information obtained from purifying the complexes using Rpd3-TAP in strains individually deleted for 11 different subunits (Table S2) [5], [7] as well as a rpd3Δ complex purified through Sin3-TAP. In order to measure the effect of the deletion on the complexes, we used the concept of information theory (*I*) based entropy (*H*), as a measure of information within a data set [16]–[18]. The *information content* is a numerical measure of disorder (i.e. unperturbed system) over order (i.e. less complex system by deleting subunits from the network). We first calculated the entropy (*H_before_*) for wild-type Rpd3-TAP and Sin3-TAP purifications (Table 1). The entropies were then calculated for each of the deletion strain purifications (*H_after_*). The effect of the deletion (*I*) was calculated by subtracting the entropy calculated in a single deletion strain (*H_after_*) from the entropy calculated in wild-type Rpd3-TAP or Sin3-TAP (*H_before_*) (Table 1). Note that only the subunits of the Rpd3 complexes were included in the entropy calculations. In principle, the higher the difference in entropies, the more information was lost after the system was perturbed and the lower the difference, the less information was lost. It is important to note that *H_after_* will decrease not only when proteins are no longer detected after gene deletion, but also when proteins' dNSAF values decrease after gene deletion. The greatest *I* value was observed when Sin3 was deleted. The next largest *I* values were obtained when Rpd3, Dep1 and Sds3 were deleted. Therefore, with the exception of Rpd3, these high differences between H_before_ and H_after_ correlate well with the numbers of subunits lost in the Sin3, Dep1 and Sds3 deletion strains (using Rpd3-TAP). It should be noted that a different TAP-tag strain had to be used for the Rpd3 deletion (i.e. Sin3-TAP instead of the Rpd3-TAP), therefore its *I* value cannot be directly compared to the I values obtained from deletion strain purifications generated with the Rpd3-TAP. These results suggest that deletions of these four proteins have the largest impact on the Rpd3 complexes, indicating their key roles for the complex integrity.

**Table 1:** Quantitative evaluation of the effect of the systematic deletion of subunits on the RPD3 complexes using Information Theory based entropy.

Proteins(a)	*H_before_* (b)	*H_after_* (c)	*I* (d)
Rpd3	2.437270	1.518056*	0.92651*
Sin3	2.444566*	0.0043	2.43297
Dep1		1.562972	0.874298
Sds3		1.573129	0.864141
Sap30		1.656705	0.780565
Rxt2		2.144370	0.2929
Pho23		2.169685	0.267585
Cti6		2.244256	0.193014
Eaf3		2.328062	0.109208
Rco1		2.257346	0.179924
Ash1		2.241281	0.195989
Ume6		2.177981	0.259289

(a) Components of the RPD3 complexes used as deletion strains;

(b) entropy calculated for the wild-type Rpd3-TAP and Sin3-TAP (*);

(c) entropy of Sin3-TAP in rpd3Δ (*) and Rpd3-TAP (rows 3–13) for each of the different 11 subunits deletion strains, as listed in ^(a)^;

(d) effect of the deletion (*I*) calculated by subtracting the entropies in the deletion strain purifications from the wild-type Sin3-TAP (*) or Rpd3-TAP (rows 3–13) entropies. A higher value of *I* indicates a strong effect of the deleted protein on the complex purification.

**Figure 3:**
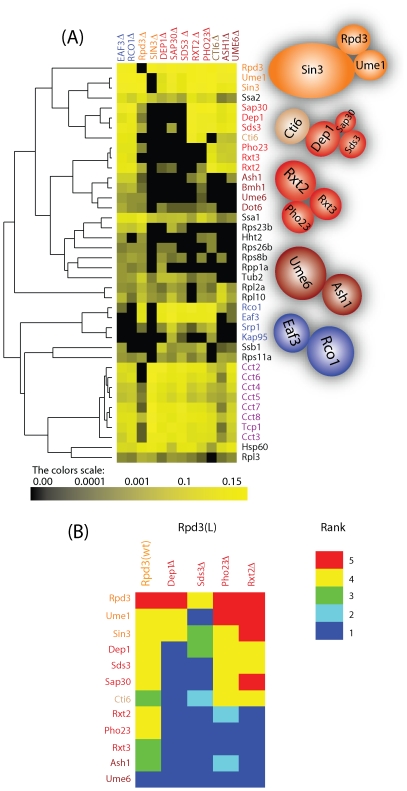
Analysis of Rpd3-TAP Deletion Strains. (A) Hierarchical clustering on the purifications of Rpd3-TAP in different deletion strains. Each *column* represents an isolated Rpd3-TAP in a different deletion strain, and each *row* represents an individual protein (prey). The color intensity represents protein abundance (dNSAF) with the brightest yellow indicating highest abundance and decreasing intensity indicating decreasing abundance. Black indicates that the protein was not detected in a particular purification. The proteins of the complexes were colored as in Figure 1. The cluster results in the formation of subcomplexes as illustrated on the right side of the cluster. (B) Ranking of the proteins within the RPD3L complex. Heat map for the protein relative abundance based rank generated from a chromatographic separation of the RPD3 complexes purified through Rpd3-TAP in four deletion strains. Red corresponds to proteins falling in a higher ranked bin (i.e. higher dNSAF) where dark blue corresponds to the lowest ranked bin (i.e. proteins were not identified in the purifications). For visualization purpose we kept only the components of the large complex.

Next, we performed a hierarchical clustering on the 12×40 matrix corresponding to the deletion dataset containing 11 deletions combined with Rpd3-TAP as well as rpd3Δ Sin3-TAP purifications (Figure 3A). We limited the analysis to 40 out of initially 80 proteins determined by SVD, since the remaining proteins were not detected in the wild-type Rpd3-TAP analysis. The result of the cluster analysis indicates a dissociation of the Rpd3 complexes through the formation of different subcomplexes. To begin, in sin3Δ Rpd3-TAP, all the proteins from the large and small complexes were not detected indicating that Rpd3L and Rpd3S might not assemble, or alternatively that Rpd3 simply cannot join the complex that is still forming in the absence of Sin3. On the other hand an analysis of rpd3Δ Sin3-TAP contained all components of the small and large complexes except Cti6 and Rxt3, even though the level of the remaining subunits was diminished (Figure S4). This suggests that Sin3 is either the scaffold for the module and therefore, in the absence of Sin3, RPD3L and RPD3S complexes are unable to assemble or, alternatively, that Rpd3 no longer associates with the complex that is still forming in the absence of Sin3.

In addition to the core complex components, other proteins that were not recovered in the sin3Δ Rpd3-TAP purification were Srp1, Kap95, Hht2, Dot6, and Bmh1. These five proteins are likely specific interactors to the Rpd3L and/or Rpd3S complexes. On the other hand, chaperones (TCP1 complex and Ssa proteins), ribosomal proteins and tubulin were still detected in the sin3Δ -Rpd3-TAP purification, which indicate that these proteins were interacting with the free TAP-tagged Rpd3, but not with the whole complex. The presence of the TCP-1 ring complex in Rpd3-TAP purifications but not in purifications using any of the other subunits as baits is in agreement with previous studies in mammals since it has been shown that the interaction of TCP-1 with HDAC3, a homolog to yeast Rpd3, is required for the proper folding of HDAC3 [19].

In all other deletions, Rpd3, Sin3, and Ume1 remained and these three proteins were in close proximity on the dendogram confirming that they form a module in both the large and small complexes (Figure 3A). The components of the small complex, Eaf3 and Rco1 along with Srp1 and Kap95, which form a dimer involved in nuclear import, were identified at similar relative abundance levels suggesting their strong association (Figure 3A). Interestingly, the components of the large complex were separated into different subcomplexes based on relative abundance similarities. One group contained Dep1, Sap30, Sds3, Cti6, another group contained Pho23, Rxt2 and Rxt3, and the third group contained Ume6, and Ash1 along with the 14-3-3 protein Bmh1 (Figure 3A), recently shown to interact with Rpd3 during S phase after HU treatment [20].

### Probabilistic deletion network and protein complex organization

**Figure 4:**
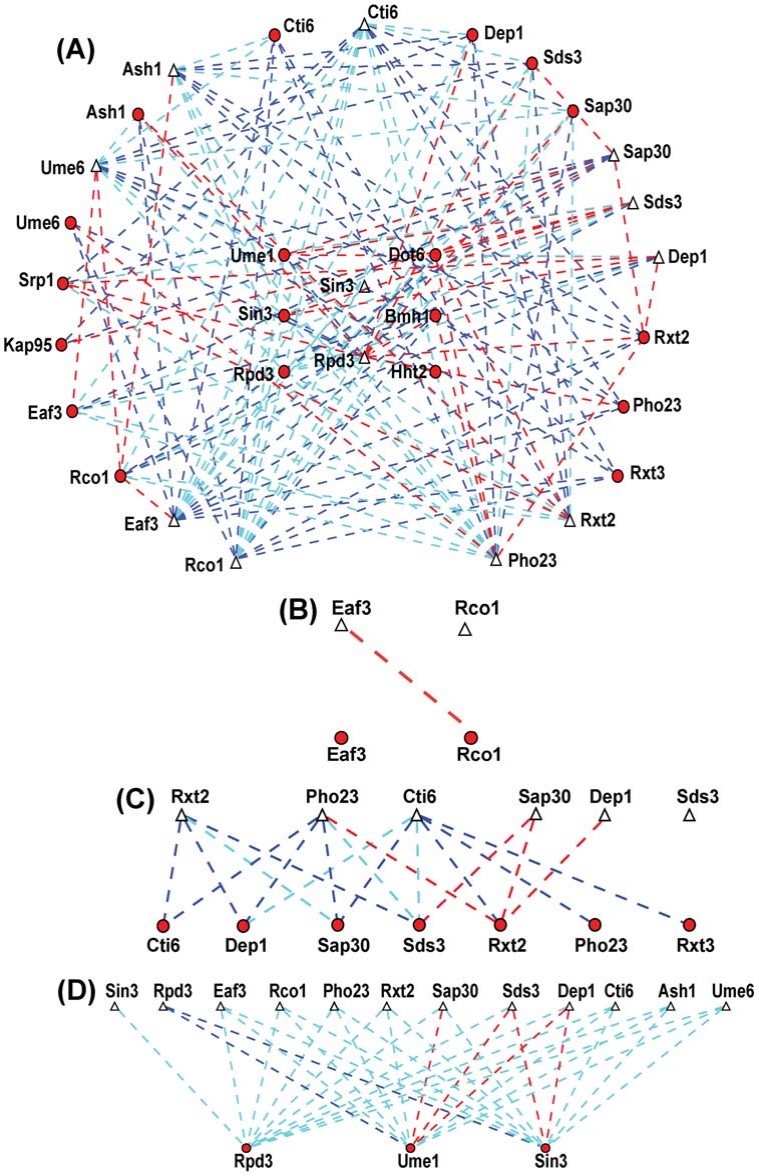
Probabilistic deletion network of the Rpd3 complexes. (A) The probabilistic protein network of the RPD3 complexes is generated by representing proteins as nodes (Rpd3-TAP in a deleted subunit are depicted by triangles and preys as circles), connected by weighted edges denoting the calculated probabilities. Blue dashed lines symbolize interactions with high probability, cyan dashed lines interactions with moderate probability, and red dashed lines interactions with low probability. (B–D) Focused probabilistic protein networks of the small complex (B), the shared module (C), and the large complex (D). Weighted edges are color-coded as in A. The Cytoscape software environment [33] was used to generate the probabilistic protein networks. For the visualization purpose, only the components of the complexes along with Srp1, Kap95, Hht2, Bmh1 and Dot6 were retained.

We assumed that each subunit in a subcomplex interact with at least one other subunit in the same subcomplex. Hence when one of the components of such pairs is deleted from the complex, the other subunit(s) should be either not recruited or maintained with a lower probability of interaction. To test this hypothesis, we used the deletion information to generate a probabilistic deletion network of the Rpd3 complexes by calculating the probability of the prey to interact with Rpd3-TAP, after sequential deletion of 11 subunits (Figure 4 and Table S4). As previously described [3], each pair of proteins (Rpd3-TAP and a prey in a deletion strain) received a probability computed from the observed experimental distributed spectral counts values using a Bayesian approach. In contrast to the wild-type network where all bait-prey pairs of the members of the complex received similar values, in the deletion network, significant differences in probabilities could be observed (Table S4). In principle, in a single purification, those preys that retain a high probability with Rpd3-TAP are expected to directly (or indirectly through the remaining proteins in the Rpd3-TAP purification) interact with Rpd3-TAP independent of the deleted subunit whereas the interaction of the absent or low probability preys with Rpd3-TAP depends on the subunit that was deleted.

The probabilistic deletion network and hierarchical clustering diagrams provide intriguing insight into the relationship and dependency between the proteins as well as the organization of the protein complexes. As mentioned before, Sin3, Rpd3, and Ume1 form a module that is shared between both the large and small complexes, for which Sin3 could act as a scaffold of this module. To begin with the small complex, in rco1Δ and eaf3Δ, Eaf3 is not detected in either, but Rco1 is present in eaf3Δ, with a lower dNSAF value (Figure 3) and diminished probability (Figure 4). This suggests that Rco1 recruits Eaf3 to the small complex, and Eaf3 helps stabilize Rco1 in the small complex. Next, when considering the Rpd3L subunits, in dep1Δ and sds3Δ, most of the components unique to the large complex are not detected, suggesting that Dep1 and Sds3 proteins are key components in the organization of the large complex (Figure 3 and 4). Even the module was affected in dep1Δ and sds3Δ by exhibiting lower probabilities than in wild-type and in the other deletion strains, indicating that the interaction of Rpd3-TAP with the module components is higher in the presence of both Dep1 and Sds3 (Figure 4). This observation is supported by the fact that the deletion of both Dep1 and Rpd3 lead to similar phenotypes (i.e. enhanced teleomeric silencing and derepressed INO1) [7].

To further decipher the relationship between these subunits, we examined another proteomic dataset generated from the chromatographic fractionation of the Rpd3L and Rpd3S complexes isolated from wild-type and four deletion strains followed by Rpd3-TAP purification (Table S3) [5]. In this dataset, Dep1 is diminished in ranking in the Sds3 deletion, Sds3 is not detected in the Dep1 deletion, and both proteins have higher ranks (see Methods) in Rxt2 and Pho23 deletions (Figure 3B) suggesting that indeed Dep1 depends on Sds3 to interact with Rpd3-TAP and not on Rxt2 or Pho23. Also it can be observed that Ume1 is not detected in sds3Δ of the Rpd3L complex (Figure 3B). In addition, Ume1 was affected more in the Dep1 deletion than in the Rxt2 and Pho23 deletions (Figure 3B). Furthermore, from the probabilistic deletion network analysis, Ume1 has the lowest probability with Rpd3-TAP in the Sds3 and Dep1 deletions. These results indicate that Sds3 helps stabilize Ume1 in the large complex. In summary, the data suggests that while Sin3 could be the scaffold for the module present in both protein complexes, Dep1 and Sds3 are key proteins in the assembly of the large complex.

**Figure 5:**
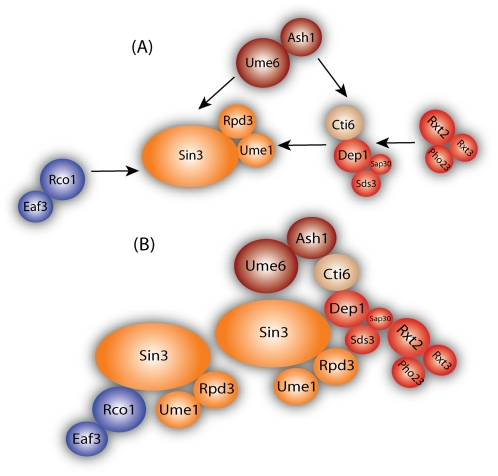
Assembly of the RPD3 complexes. (A) We positioned all subcomplexes next to the module based upon above observations, however we placed the first subcomplex in closer proximity of the module since its members affected the stability of the module. (B) Final assembled complex. Red proteins correspond to the proteins in the RPD3L, blue corresponds to the proteins in the RPD3S and the shared module was colored in orange. The two sequence specific repressors Ash1 and Ume6 are colored in dark red and Cti6 in light brown. The size of the inset circles corresponds to the molecular weights of the proteins illustrated.

In sap30Δ, only one protein of each subcomplex, Sds3 and Rxt2, are present at a lower probability with Rpd3-TAP (Figure 4), indicating that those proteins might be the connection to the module. The protein Cti6 appears to have a direct or indirect (through other absent proteins) connection with Dep1, Sap30 and Sds3 in the large complex since it was not detected in these deletion strains. This can be also observed from the ranked deletion matrix (Figure 3B) where Cti6 was not detected in dep1Δ and was lower in sds3Δ compared to rxt2Δ and pho23Δ. Regarding the second subcomplex, only Rxt2 was present with a lower probability in pho23Δ while Pho23 and Rxt3 were absent from all other subcomplex deletions, indicating that Rxt2 is the protein that brings Rxt3 and Pho23 to the complex. Finally, in cti6Δ, all the components of the large complex were present except for Ash1 and Ume6. Based on this, we believe that Ash1 interacts with Cti6 to recruit the Rpd3L complex to carry out its function as sequence specific repressor [5]. Based on the deletion network results, we propose a protein interdependency-interaction model of the Rpd3 complexes (Figure 5). We positioned all subcomplexes next to the module based upon the above observations, however we placed the first subcomplex in closer proximity to the module since its members affected the stability of the module (i.e., lower probabilities in deletion strains with Rpd3-TAP).

A number of lines of evidence support our model. First, in yeast, we determined whether our results correlated to the effect of the different Rpd3 complex components on gene transcription. To achieve this, we compared our results with a genome-wide microarray analysis performed on Rpd3 complex in 11 deletion strains by Keogh et al. [6]. Interestingly, within the two complexes, proteins identified to be in proximity within the same subcomplex or to be members of the small complex based upon our analysis showed similar effects on gene expression (i.e., the expression profiles of the deletion strains cluster together). For instance, Eaf3 and Rco1, components of the Rpd3S complex, tightly cluster based on the correlation of its expression profiles [6]. Also, deletions of Dep1 and Sds3 exhibited a similar effect on the global gene expression profiles followed by Sap30, an order of severity that is identical to our deletion network analysis [6]. The loss of Rxt2 and Pho23 lead to the same effect on the gene profiles, again suggesting that they are in the same module. Lack of Rpd3 and Sin3, two proteins that were identified as being tightly associated in the current study, also led to very similar global gene expression changes. Additional studies support the potential role of Sin3 as the scaffold for both the large and small complexes. Yeast and mammalian Sin3 have four paired amphipathic helix (PAH) motifs [21]–[24]. Mammalian studies have shown that these PAH motifs are the domains in Sin3 with which other proteins bind [25], [26]. Our results agree with these previous studies that suggest that Sin3 is the scaffold for the Rpd3 complexes.

## Discussion

Our results demonstrates that for a stable complex such as Rpd3, the wild-type protein network assembled using TAP-tag approaches allows to determine the subunits of the complex as well as *bona fide* protein interactions, but not the connectivity between the proteins. However, perturbation of the complex by genetic deletion of several subunits resulted in its dissociation into subcomplexes. After determining the effect of each deletion by calculating the information theory based entropy, we generated a probabilistic deletion network for the Rpd3-TAP purified deletion mutants. We showed, that unlike the wild-type network, after the deletions, the obtained probabilities represent the direct relationship between deleted components and preys. The conjunction of the hierarchical clustering analysis and this probabilistic deletion network derived from quantitative data resulted in a model for the Rpd3 complexes.

Knowing how proteins associate into a complex, in particular in the case of less-characterized complexes, could provide valuable insights into the function of its components. Strongly associated components of a complex might exert the same or similar activities and/or might depend on each other to operate properly. Indeed, we observed that there is a correlation between the proximity of two proteins in the Rpd3 complexes and its function, i.e. specific repression of transcription as measured by the effect on genome-wide gene expression [6]. The protein network that we proposed for the Rpd3 complexes is highly relevant for both basic and applied research. In particular, the two mammalian proteins BRMS1L and mSds3, which are the orthologs to the Rpd3 components Dep1 and Sds3, are known to contribute to the suppression of metastasis and proper embryonic development [27]–[30].

In summary, we demonstrate that the protein connectivity of a protein complex can be determined from quantitative proteomics data generated from a deletion network analysis. In such an analysis a wild type and deletion network needs to be generated. This straightforward approach can be used on a wide variety of protein complexes that contain a number of nonessential subunits in genetically tractable organisms where knockouts can be made like *S. cerevisiae*, *S. pombe*, *D. melanogaster*, and *C. elegans* for example. The objective is to generate quantitative deletion-interaction maps that will provide valuable insights into the function of the proteins as well as in discerning subcomplexes within protein complexes.

## Materials and Methods

### Identification of proteins by MudPIT

The *Saccharomyces cerevisiae* Rpd3-TAP and TAP-Dep1 strains were cloned, expressed and purified as previously described [5], [7]. The remaining TAP tagged proteins (Sin3, Eaf3, Rco1, Sap30, Pho23, Cti6, Rxt2, Ash1 and Ume6) were purchased from Open Biosystems and purified as reported [7]. The null mutant strains (sin3Δ, eaf3Δ, rco1Δ, dep1Δ, sap30Δ, pho23Δ, cti6Δ, rxt2Δ, ash1Δ, and ume6Δ) were purchased from Open Biosystems, amplified by PCR and transformed into an Rpd3-TAP strain in a w303 or BY4741 background. Rpd3Δ was similarly obtained and transformed into a Sin3-TAP strain in a BY4741 background. Identification of proteins was accomplished by Multidimensional Protein Identification Technology (MudPIT) as previously described [9]. dNSAFs were calculated based on distributed spectral counts, in which shared spectral counts were distributed based on spectral counts unique to each isoform. The dNSAF equation takes into account the spectral counts of shared peptides, i.e. peptides that are present in more than one protein, and distributes these spectral counts based on a distribution factor, *d*, where:
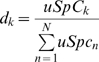



That is, *d* equals the number of unique spectral counts (*uSpC*) from a given protein *k* divided by the total number of *uSpC* from all *n* proteins with which protein *k* shares peptide(s). Therefore, a fraction of the shared spectral counts (*sSpC*) are distributed amongst the unique proteins and each shared spectral count is counted once and once only.

The dNSAF of a given protein, *k*, is then defined by the following equation where L is the length of the protein:
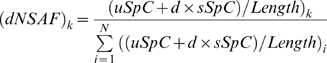



To ensure the reproducibility of the data we calculated the Pearson correlation for each pair of replicates using the dNSAF value of each subunit in the complex (Table S6). We defined a good replicate if the Pearson correlation coefficient was greater than 0.5, and this information is provided in more detail in the Supporting Information. In addition, we also performed hierarchical cluster analysis using replicates of several selected wild-type baits. The results showed that the small variations in dNSAF values among the replicates do not alter the output of the hierarchical cluster analysis for the known complexes, further indicating the reproducibility of the data set (Figure S5).

#### Entropy

In thermodynamics, entropy has important physical implications as the amount of “disorder” of a system. In information theory, the quantity entropy plays a central role as a measure of information. To quantitatively characterize the effect of the deletion on the complexes, we utilize the Shannon entropy defined by:
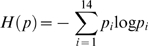
(1)were *p_i_* is the dNSAF of a prey in a given bait, and the summation is taken over all of the non-zero *p_i_* (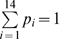). Only the subunits of the complexes were included in the analysis. The *information* is calculated based on the entropy as follows:
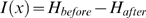
(2)


### Hierarchical clustering and Probabilistic Network Analysis

Relative protein abundances represented as dNSAF values were clustered using the Pearson correlation as a distance metric and UPGMA as a method using PermutMatrix software [31]. As previously described [3], each pair of proteins (Rpd3-TAP in a deleted subunit and a prey) received a probability, computed from the observed experimental distributed spectral counts values using a Bayesian approach. Network analysis was largely carried out as described previously [3], with modifications as described below.

#### Singular Value Decomposition (SVD)

To determine the proteins enriched in the purifications (i.e. Rpd3 complexes), we applied singular vector decomposition (SVD) on the wild-type matrix (11 baits×429 prey proteins) with the matrix element representing the normalized spectral count, i.e. dNSAF, for each prey and bait as previously described [3]. We used the information obtained from the first left singular vector (lsv) to define the proteins that are enriched from the purifications by using a rank estimated method. Furthermore, we investigated the distribution of the lsv by plotting the components of the first left singular vector in a log-log and a linear-log scale (Figure S3) and observed that the data is characterized by a double exponential. We also found that the majority of the core components of the complex are situated on the first exponential.

The subunits of the Rpd3 complexes are all founded in the top 21 (first exponential) except Ume6 protein which was situated at the beginning of the second exponential. In addition to the subunits of the complex, eight new proteins were coming at the top (first exponential) (Bmh1, Srp1, and four Hsp70 chaperone homologs (Ssa and Ssb). The rest of the components of the first left singular vector are plotted in a linear-log scale with an exponential fitting. Therefore, in this analysis, proteins were retained if their corresponding coefficients were larger than a cutoff of ∼0.004. This cutoff was chosen to ensure the inclusion of all known Rpd3/Sin3 components (including Ume6 which was founded at a substoichiometric level compared with the rest of the components of the Rpd3 complexes), resulting in a total of 80 proteins which comprise the most essential proteins in the dataset as well as new candidate proteins.

### Probabilistic analysis of the deletion network

In this section, we describe a probabilistic method for calculating the connectivity between a Rpd3-TAP (bait) in a deletion subunit and a prey protein. The observed spectral counts of each prey in the baits were used to compute the following probabilities.

To quantify the connectivity relationship between a prey protein *i* (*i* = 1,…, *N*) and a bait *j* (*j* = 1,…, *M*), we first estimated the conditional probability, that is the probability of a prey being in the sample given the fact that the bait j is in the sample, by:
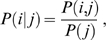
(3)where *P*(*i,j*) is the joint probability between protein *i* and bait *j* and is defined as:
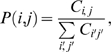
(4)where 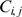 is the number of distributed spectral abundance factor (i.e. spectral counts divided by protein's length) value of prey i in bait j while 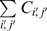 sums the total number of distributed spectral abundance factor. *P*(*j*) is the likelihood of bait *j* and is estimated by:
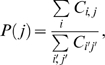
(5)where 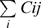 sums the distributed spectral abundance factor values of preys, *i* in the bait, *j*. When the conditional probability is known, we can calculate the marginal probability of prey *i* using:
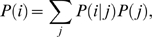
(6)where the summation is over all possible values of *j*.

For a bait, *j* and prey, *i*, the posterior probability *P*(*j|i*) defined by Bayes' rule:
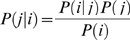
(7)quantifies the preference of a bait (Rpd3-TAP) to have a connection with a prey. Similar to previous studies, an uninformative prior probability was chosen, that is, 1/N. Since probabilities correlate to the tendency of two proteins to associate with each other, we partition the components of the Rpd3 complexes into three biologically meaningful groups, i.e. those exhibiting low (less than 0.02), medium (0.02–0.04) and high (greater than 0.04) probabilities with Rpd3-TAP (Figure 3). In principle, in a single purification, those preys that retain a high probability with Rpd3-TAP are expected to directly (or indirectly through the remaining proteins in the Rpd3-TAP purification) interact with Rpd3-TAP independent of the deleted subunit, whereas the interaction of the absent or low probability preys with Rpd3-TAP depends on the subunit that was deleted.

#### Re-arrangement of data for the Rpd3L complex into bins based on abundance levels

To underline the effect of the deletion on the subunits of the complex, we binned the data according to their abundance level. Binning is an established approach for ranking data which has been used in many biological applications [32]. The entire normalized dataset extends over three orders of magnitude (0.1 to 0.001). The 5 bins were created as follows: bin 1: 0.1 to 0.04; bin 2: 0.039−0.01; bin 3: 0.01−0.004, and bin 4: 0.0039−0.001. All remaining proteins which were not detected in the purifications were included in bin 5. The abundances of the proteins in the matrix were replaced by their corresponding ranking values.

## Supporting Information

Figure S1 General strategy for assembling intensity based local protein interaction network. Eleven unique bait proteins were TAP tagged and their respective protein interactions determined by multidimensional protein identification technology. For each identified non-redundant protein, spectral counts were converted to the distributed normalized spectral abundance factors. After mathematically removing contaminants, the top 80 ranked proteins were retained and subject to hierarchical clustering analysis. To determine the relationship between proteins within the complex, the components of the small and large complexes were systematically deleted from the network through the purification of Rpd3-TAP in a deletion strain as described in the main text. A hierarchical cluster analysis was performed on the dNSAF values. The result of the cluster indicates a dissociation of the RPD3S and RPD3L complexes through the formation of different subcomplexes. The complexes were also disrupted using fractionation of the RPD3L and RPD3S complex by chromatography as explained in the text. The proteins were sorted and ranked based on the dNSAF values (due to the small size the cluster could not be performed) and the constructed deletion matrix was therefore used to determine the association between the subunits of the complexes. To measure the effect of the deletion, the information based entropy was computed. Finally, a Bayesian analysis of the distributed spectral abundance factor deletion information on a per bait basis resulted in a network that reflects the probability between Rpd3-TAP in a subunit deletion strain (or bait) and prey interaction. The entropy, the deletion cluster, the ranked proteins in the deletion matrix, and the deletion network were used in the assembly of RPD3 complex model. Genetic information was used to validate the relationship between the components of the large and small complexes.
(0.64 MB PDF)

Figure S2 Distributions of the left singular vector (lsv). We used the information obtained from the first left singular vector (lsv) to define the proteins that are enriched from the purifications by using a rank estimated method. Furthermore, we investigated the distribution of the lsv and observed that the data is characterized by a double exponential and found that the majority of the core components of the complex are situated on the first exponential. (A) The components of the first left singular vector are plotted in a log-log scale (B) The components of the first left singular vector are plotted in a linear-log scale (C) The top 21 components of the left singular vector corresponding to the first 21 highly abundant proteins are plotted in the linear-log scale indicating an exponential behavior. The subunits of the RPD3 complexes are all founded in the top 21 except for the Ume6 protein. In addition to these proteins, eight new proteins were coming at the top (Bmh1, Srp1, and four Hsp70 chaperone homologs (Ssa and Ssb). (D) The rest of the components of the first left singular vector are plotted in a linear-log scale with an exponential fitting.
(0.32 MB PDF)

Figure S3 Hierarchical clustering on the Jaccard indices. A symmetrical matrix (11×11) consisting of Jaccard values calculated for each bait pair was hierarchical clustered. The color intensity represents Jaccard index with the brightest yellow indicating highest index and decreasing intensity indicating decreasing index.
(0.24 MB PDF)

Figure S4 Sorted relative abundances of the Rpd3/Sin3 components. The dNSAF values for subunits of the small and large complexes were plotted in (A) rpd3Δ Sin3-TAP and (B) Sin3-TAP wild-type background.
(0.27 MB PDF)

Figure S5 Hierarchical cluster analysis of the wild-type data set including replicates for several baits. Each column represents an isolated purification, and each row represents an individual protein (prey). Several replicates were included in the cluster analysis (depicted by ‘_R’). The color intensity represents protein abundance (dNSAF) with the brightest yellow indicating highest abundance and decreasing intensity indicating decreasing abundance. Black indicates that the protein was not detected in a particular purification. The proteins of the complexes were colored as in Fig. 1.
(0.27 MB PDF)

Table S1 List of Proteins Detected in S. cerevisiae Rpd3/Sin3 Wild-Type Datasets Prior to Contaminant Extraction.
(1.23 MB XLS)

Table S2 List of Proteins Detected in S. cerevisiae Rpd3-TAP or Sin3-TAP Deletion Datasets.
(1.52 MB XLS)

Table S3 List of Proteins Detected in the Separated Rpd3S and Rpd3L Deletion Datasets.
(0.61 MB XLS)

Table S4 List of protein-protein interactions within Rpd3 wild-type and deletion networks.
(0.12 MB XLS)

Table S5 List of Protein Complexes Detected in S. cerevisiae Rpd3 wild-type Dataset.
(0.03 MB XLS)

Table S6 Supporting results and Table S6
(0.05 MB DOC)
