# Supplementary figures and images for: Determining Protein Complex Connectivity Using a Probabilistic Deletion Network Derived from Quantitative Proteomics
Source: PLoS One. 2009 Oct 6;4(10):e7310. doi: 10.1371/journal.pone.0007310 (PMC2751824; doi:10.1371/journal.pone.0007310)

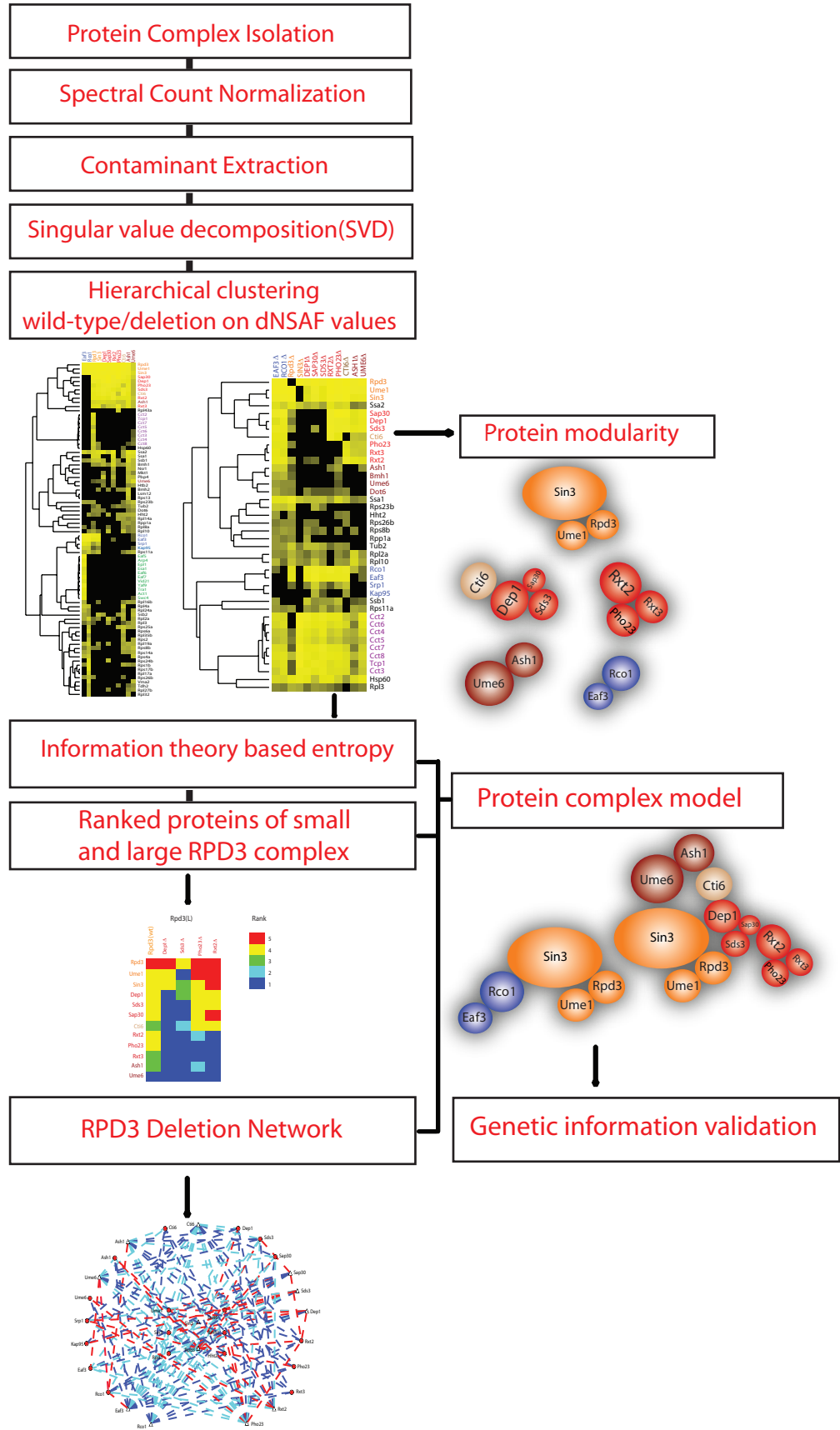

Supplement: Figure S1 — General strategy for assembling intensity based local protein interaction network. Eleven unique bait proteins were TAP tagged and their respective protein interactions determined by multidimensional protein identification technology. For each identified non-redundant protein, spectral counts were converted to the distributed normalized spectral abundance factors. After mathematically removing contaminants, the top 80 ranked proteins were retained and subject to hierarchical clustering analysis. To determine the relationship between proteins within the complex, the components of the small and large complexes were systematically deleted from the network through the purification of Rpd3-TAP in a deletion strain as described in the main text. A hierarchical cluster analysis was performed on the dNSAF values. The result of the cluster indicates a dissociation of the RPD3S and RPD3L complexes through the formation of different subcomplexes. The complexes were also disrupted using fractionation of the RPD3L and RPD3S complex by chromatography as explained in the text. The proteins were sorted and ranked based on the dNSAF values (due to the small size the cluster could not be performed) and the constructed deletion matrix was therefore used to determine the association between the subunits of the complexes. To measure the effect of the deletion, the information based entropy was computed. Finally, a Bayesian analysis of the distributed spectral abundance factor deletion information on a per bait basis resulted in a network that reflects the probability between Rpd3-TAP in a subunit deletion strain (or bait) and prey interaction. The entropy, the deletion cluster, the ranked proteins in the deletion matrix, and the deletion network were used in the assembly of RPD3 complex model. Genetic information was used to validate the relationship between the components of the large and small complexes. (0.64 MB PDF) [file pone.0007310.s001.pdf]

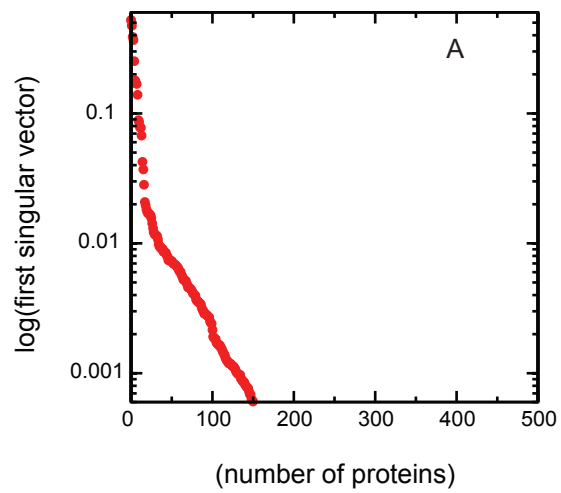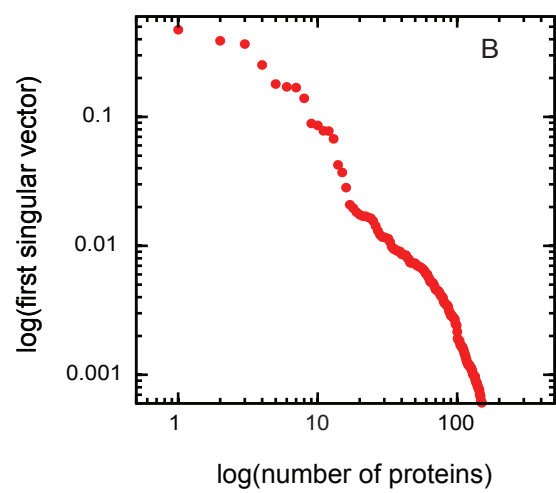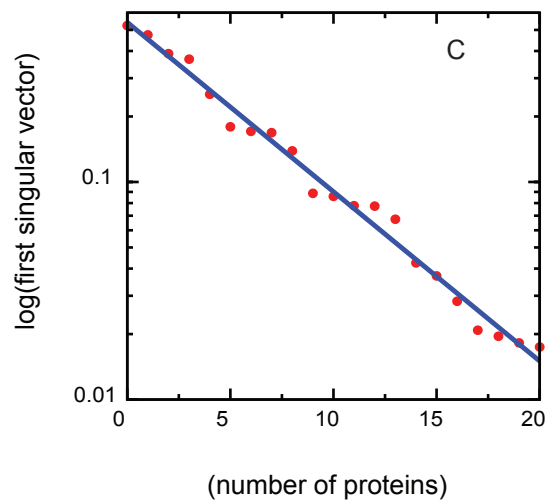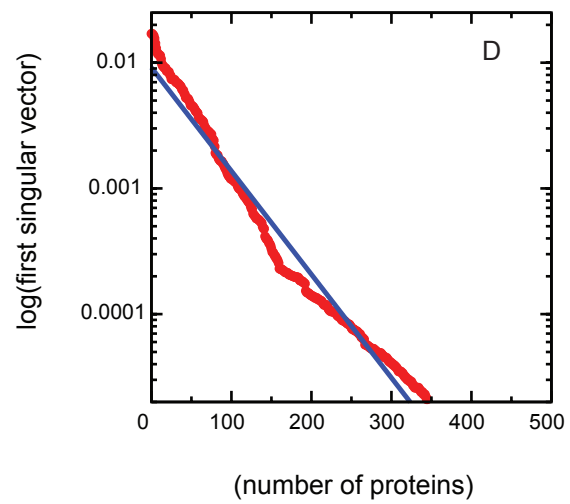

Supporting Figure 3. Sardiù et al.

Supplement: Figure S2 — Distributions of the left singular vector (lsv). We used the information obtained from the first left singular vector (lsv) to define the proteins that are enriched from the purifications by using a rank estimated method. Furthermore, we investigated the distribution of the lsv and observed that the data is characterized by a double exponential and found that the majority of the core components of the complex are situated on the first exponential. (A) The components of the first left singular vector are plotted in a log-log scale (B) The components of the first left singular vector are plotted in a linear-log scale (C) The top 21 components of the left singular vector corresponding to the first 21 highly abundant proteins are plotted in the linear-log scale indicating an exponential behavior. The subunits of the RPD3 complexes are all founded in the top 21 except for the Ume6 protein. In addition to these proteins, eight new proteins were coming at the top (Bmh1, Srp1, and four Hsp70 chaperone homologs (Ssa and Ssb). (D) The rest of the components of the first left singular vector are plotted in a linear-log scale with an exponential fitting. (0.32 MB PDF) [file pone.0007310.s002.pdf]

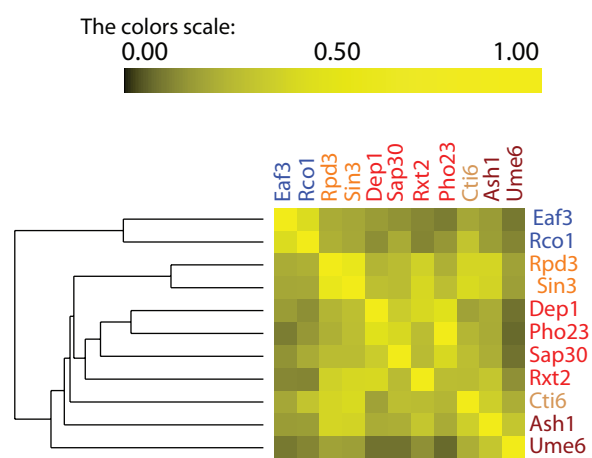

Supporting Figure 2. Sardi et al.

Supplement: Figure S3 — Hierarchical clustering on the Jaccard indices. A symmetrical matrix (11×11) consisting of Jaccard values calculated for each bait pair was hierarchical clustered. The color intensity represents Jaccard index with the brightest yellow indicating highest index and decreasing intensity indicating decreasing index. (0.24 MB PDF) [file pone.0007310.s003.pdf]

A

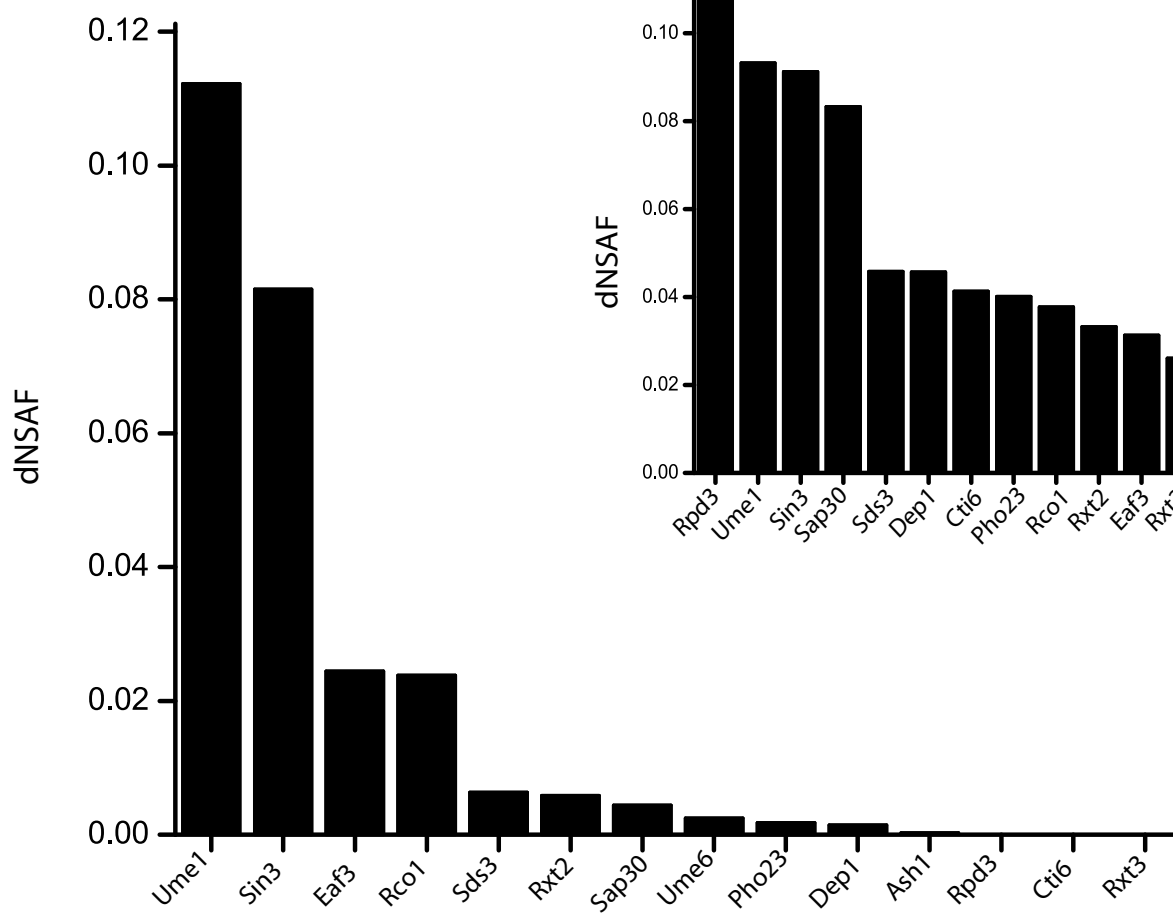

B

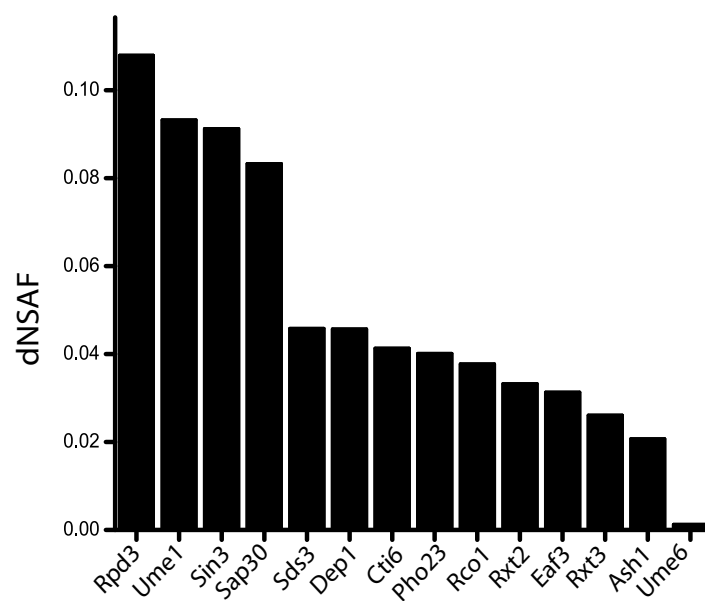

Supplement: Figure S4 — Sorted relative abundances of the Rpd3/Sin3 components. The dNSAF values for subunits of the small and large complexes were plotted in (A) rpd3Δ Sin3-TAP and (B) Sin3-TAP wild-type background. (0.27 MB PDF) [file pone.0007310.s004.pdf]

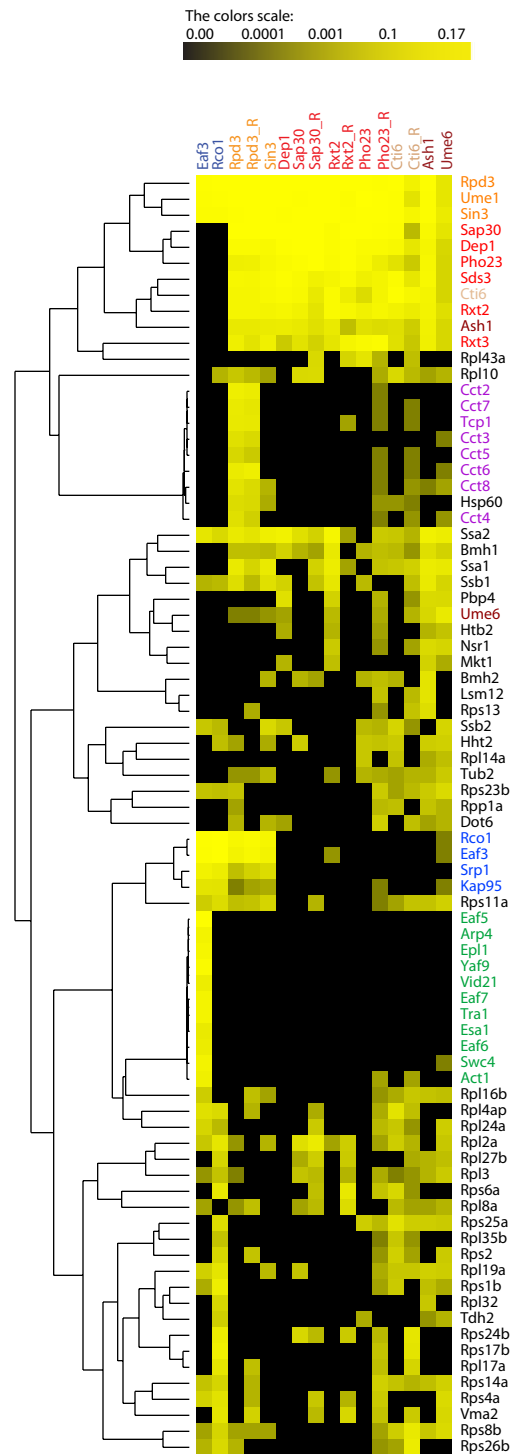

Supporting Figure 5. Sardi et al.

Supplement: Figure S5 — Hierarchical cluster analysis of the wild-type data set including replicates for several baits. Each column represents an isolated purification, and each row represents an individual protein (prey). Several replicates were included in the cluster analysis (depicted by ‘_R’). The color intensity represents protein abundance (dNSAF) with the brightest yellow indicating highest abundance and decreasing intensity indicating decreasing abundance. Black indicates that the protein was not detected in a particular purification. The proteins of the complexes were colored as in Fig. 1. (0.27 MB PDF) [file pone.0007310.s005.pdf]
