# Supplementary material for: Determining Protein Complex Connectivity Using a Probabilistic Deletion Network Derived from Quantitative Proteomics
Source: PLoS One. 2009 Oct 6;4(10):e7310. doi: 10.1371/journal.pone.0007310 (PMC2751824; doi:10.1371/journal.pone.0007310)
Supplement: Table S6 — Supporting results and Table S6 (0.05 MB DOC) [file pone.0007310.s011.doc]

**Supporting Information for**

**Determining Protein Complex Connectivity using a Probabilistic Deletion Network Derived from Quantitative Proteomics**

Mihaela E. Sardiu^1^, Joshua M. Gilmore^1^, Michael J. Carrozza^2^, Bing Li^3^, Jerry L. Workman^1^, Laurence Florens^1^, and Michael P. Washburn^1,4^

^1^Stowers Institute for Medical Research, Kansas City, MO 64110

^2^Laboratory of Structural Biology, National Institute of Environmental Health Sciences, Research Triangle Park, NC 27709

^3^Department of Molecular Biology, UT Southwestern Medical Center, Dallas, TX 75390

^4^To whom correspondence should be addressed

Michael P. Washburn

Stowers Institute for Medical Research

1000 E. 50^th^ St.

Kansas City, MO 64110

Phone: 816-925-4457

Fax: 816-926-4694

e-mail: mpw@stowers-institute.org

**Supporting Results**

***Analysis of the wild-type histone deacetylase complex.***

The remaining proteins after SVD analysis, which can be considered as proteins specifically binding to each of the baits (i.e. binding to the known components of the Rpd3 complexes), were next subjected to a more in detail analysis. Without prior restriction to the complex, we analyzed the whole dataset in order to determine if the additional low abundant proteins, which were not considered to be known or novel components of the Rpd3L and Rpd3S, might be shared among multiple baits or if those proteins represent interactions outside the Rpd3 complexes. In order to achieve this, we first determined the similarity of the proteins present in each bait by calculating the Jaccard indices between all combinations of the purifications and applied hierarchical clustering on the resulting Jaccard matrix. The results obtained from this analysis led to three clusters corresponding to the small complex, the large complex, and to the two sequence specific repressors Ash1 and Ume6 (Figure S3). Although the hierarchical cluster analysis was able to separate the small complex from the large complex, the indices were ranging from low to medium values (in contrast, if Jaccard indices were calculated only with true components of the Rpd3 complexes, high indices were obtained). For instance, a low index of 0.390625 was found for the Eaf3 and Rco1 bait pair (the highest value obtained for the Eaf3 bait compared with the rest of the baits) and a medium index of 0.541176 (the highest value obtained from the Jaccard analysis) was found between Rpd3 and Sin3. This result indicated that except for the subunits of the Rpd3 complexes, the majority of the proteins are specific to each of the baits (i.e. present only in a single bait).

We next identified the specific interactions to each of the baits (i.e. present only in a single purification) in the small and large complex. For the baits belonging to the small complex, we found that components of the NuA4 complex (Act1, Tra1, Vid21, Epl1, Eaf5, Arp4, Eaf7, Yaf9, Esa1, Yng2, and Eaf6) were present only in the Eaf3 purification, which is expected since the protein Eaf3 is shared between the RPD3 small complex and the NuA4 complex; Rco1 bait co-purified 3 unique proteins, of which all were ribosomal proteins (Rpl4b, Rpl17a, and Rps17b). In the module, Rpd3 as a bait pulled eight unique proteins, four of which are chaperones (Cct5, Tcp1, Cct7, and Cct2), one protein of unkown function (Ecm5), Snt2, Smc5 and Yjl010c. Three proteins were unique to the Sin3 purification, i.e. Opi1, His3, and Rps18b. In the large complex, only in the Dep1 purification non-complex proteins were pulled that were shared by the other purifications. Three unique proteins (Kar4, Not3 and Ygl226w) were founded in the Rxt2 bait, two proteins were unique to Pho23 (Yor112w and Rpl43a), four proteins were unique to the Sap30 purification (Adh3, Pdc5, Ycf1, and Pgk1), and seven proteins were unique to the Cti6 purification (Gcn20, Rps30b, Rpl9a, Nop12, Rpl8b, Psl1, and Nsp1). For the transient protein Ume6 a total of 220 unique proteins were identified. This is expected, since Ume6 is known to be involved in different processes. In order to further characterize those proteins that were only purified with Ume6, we used Gene Ontologies and identify biological processes that were significantly overrepresented (P<10^-4^) among the proteins in the Ume6 bait. According to this analysis, a few classes were enriched such as metabolism (RNA metabolic process; nucleobase, nucleoside, nucleotide and nucleic acid metabolism), transcription, cell organization and biogenesis (DNA organization; chromatin architecture maintenance; and RNA-protein complex biogenesis), suggesting that those co-purified proteins have a similar function as Ume6. However, for the other bait representing a transient protein, Ash1, six proteins (Krr1, Cka2, Imd2, Dpb6, Lsm12, and Rps13) were unique. Finally, all the proteins identified in the wild-type dataset were separated into the complexes (134 protein complexes were detected by SGD Gene Ontology Slim Mapper) and reported in Table S5.

***Analysis of the histone deacetylase complex in a Rpd3-TAP deletion.***

We next investigated the deletion dataset in order to determine whether the deletion of subunits of the complexes affect the association of non-complex proteins with a bait, i.e. whether any of the non-complex proteins are enriched in the deletion strains and accordantly potentially interact with the bait. Since all the deletions are obtained through Rpd3-TAP, we hence only compared the result obtained from Rpd3-TAP in all deletions strains with the wild-type Rpd3-TAP. We computed the average dNSAF of all Rpd3-TAP in 11 deletion strain and ranked the proteins accordingly. We examined the first top 50 rank proteins (with the highest dNASF average) and observed that in addition to the known components of the complexes, the rest of the proteins were mainly chaperones (Tcp1, Cct7, Cti6, Cct8, Cct6, and Cct2), components of the NuA4 complex and ribosomal proteins. However, among the 50 most abundant proteins present in the deletion strains, two other proteins that did not fall into above categories were detected at higher abundance level, i.e. Ecm5 (unkown protein) and Snt2 (DNA binding protein). Interestingly, Ecm5 and Snt2 proteins were present only in the wild-type Rpd3 purification and in the majority of deletion strains Rpd3-TAP purifications, however were absent in the rpd3Δ Sin3-TAP, strongly indicating their association to the Rpd3 are independent of all other components of the Rpd3 complexes and therefore might be of direct nature. On the other hand, both proteins exhibit only a very small number of additional proteome-wide protein interactions (4 protein interactions for Ecm5 and 3 interactions for Snt2, according to BioGrid), suggesting that this interaction to Rpd3 is important for Ecm5 and Snt2 in order to exert its function.

**Supporting Table 6. Replicates of seven wild-type purifications as well as from two different deletion strains Rpd3-TAP purifications were tested for reproducibility.** The Pearson correlation coefficient was computed for each of the replicate pair using the dNSAF value of each subunit in the complex. The high Pearson correlation coefficients indicate a good reproducibility of the data sets.

| Baits | Pearson correlation coefficients |
| --- | --- |
| Rpd3 | 0.8181 |
| Sin3 | 0.8788 |
| Sap30 | 0.9545 |
| Dep1 | 0.8357 |
| Rxt2 | 0.8287 |
| Pho23 | 0.9699 |
| Cti6 | 0.6962 |
| Rpd3-TAP eaf3Δ | 0.8752 |
| Rpd3-TAP cti6Δ | 0.8771 |
